# New sights on long non-coding RNAs in glioblastoma: A review of molecular mechanism

**DOI:** 10.1016/j.heliyon.2024.e39744

**Published:** 2024-10-23

**Authors:** Saeid Bagheri-Mohammadi, Arezoo Karamivandishi, Seif Ali Mahdavi, Ali Siahposht-Khachaki

**Affiliations:** a Department of Paramedicine, Amol School of Paramedical Sciences, Mazandaran University of Medical Sciences, Sari, Iran; b Immunogenetics Research Center, Mazandaran University of Medical Sciences, Sari, Iran; c Department of Tissue Engineering and Applied Cell Science, School of Advanced Technologies in Medicine, Shahid Beheshti University of Medical Sciences, Tehran, Iran; d Immunogenetics Research Center, Department of Physiology, Faculty of Medicine, Mazandaran University of Medical Sciences, Sari, Iran

**Keywords:** Long non-coding RNAs, Glioblastoma, Cancer, Molecular mechanism, RNAs

## Abstract

Glioma or glioblastoma (GBM) is one of the aggressive and fatal primary cerebral malignancies, with the highest mortality rate among all brain-related tumors. Also, glioma mainly progresses as a more invasive phenotype after primary treatment. Cumulative evidence suggested that dysregulation of noncoding RNAs (ncRNAs) such as long non-coding RNAs (LncRNAs) and microRNAs (miRNAs) are associated with tumor initiation, progression, and drug resistance, through epigenetic modifications, transcriptional, and post-transcriptional processes in the cells. Many scientific investigations have revealed that LncRNAs play important roles in various biological procedures linked with the development and progression of GBM. In recent years, it has been shown that dysregulation of molecular mechanisms in many LncRNAs such as MIR22HG, HULC, AGAP2-AS1, MALAT1, PVT1, TTTY14, HOTAIRM1, PTAR, LPP-AS2, LINC00336, and TINCR are connected with the GBM. Therefore, this scientific review paper focused on the molecular mechanisms of these LncRNAs in the context of GBM.

## 1 Introduction

**Fig. 1:**
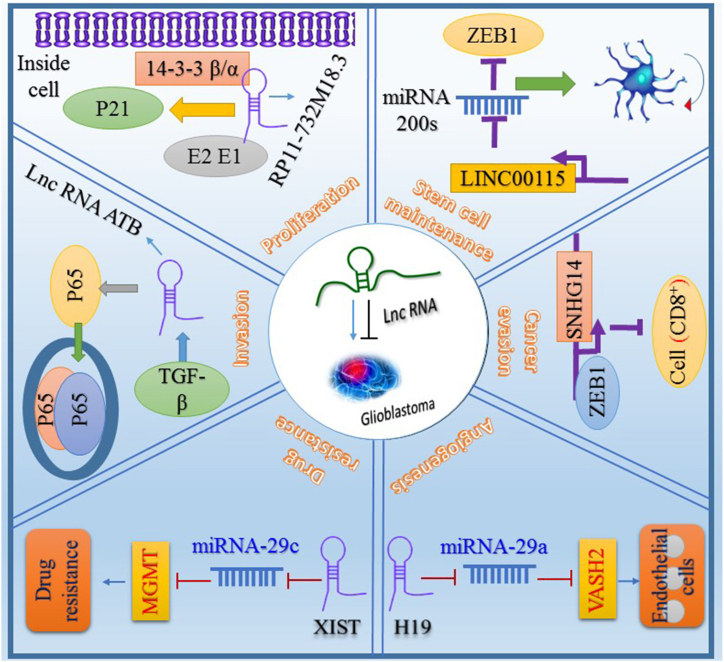
The role of noncoding RNAs (ncRNAs) in regulation of GBM. Arrows show increased activity and hammerhead arrows show decreased activity.

Long non-coding RNAs (LncRNAs) have a critical function in various physiological processes and pathological conditions including tumor formation, malignant transformation, apoptotic cell death, epigenetic modification, genomic imprinting, spermatogenesis regulation, and maturation [1,2]. Prominent advances in whole-genome sequencing have been obtained via microarray technology and next-generation sequencing methods and introduced a more comprehensive knowledge about LncRNAs. In the human genome, one-two percent of genes encode proteins, and 98 percent of the remaining encoded products are known as noncoding RNAs (ncRNAs) such as LncRNAs and microRNAs (miRNAs). Unlike miRNAs, LncRNAs can control the expression of genes at different levels including transcriptional regulation, post-transcriptional and epigenetic regulation [3,4]. It has been shown that LncRNAs have an important function in the conversion of RNAs into small interfering RNAs. According to the investigations, miRNAs can control LncRNAs, and also LncRNAs can regulate the expression levels of miRNAs, forming a two-way regulatory network with important functions. Cumulative scientific investigations revealed that LncRNAs are engaged in both individual development and a multitude of human disorders. Experimental studies indicated that Tumor formation and malignancy are correlated with the abnormal expression of LncRNAs, making these ncRNAs attractive targets to prevent human diseases [[5], [6], [7], [8], [9], [10]]. Accordingly, dysregulation of LncRNAs is correlated with GBM and it can enhance the tumor invasion and metastasis (Fig. 1). GBM is an aggressive malignant cerebral tumor and the exact molecular mechanism of this type of cancer is not clear. The increasing levels of several tumorigenic LncRNAs have been identified in GBM. It is well established that the oncogenic properties of LncRNAs are mediated by different molecular signaling pathways. In the last decades, the cumulative investigation of LncRNAs signifies novel perspectives on the molecular mechanisms implicated in GBM [[11], [12], [13]]. However, little evidence is available to demonstrate the relationship between the regulatory pathways of LncRNAs and underlying molecular mechanisms. Hence, this scientific paper is designed to discuss the various LncRNAs (MIR22HG, HULC, AGAP2-AS1, MALAT1, PVT1, TTTY14, HOTAIRM1, PTAR, LPP-AS2, LINC00336, TINCR, etc.) molecular pathways involved in GBM. It is crucial to have a deep comprehension of these molecular pathways to advance effective therapeutic strategies.

## 2 Non-coding RNAs in cancer

Tumor formation and progression is a lethal disorder with increasing worldwide mortality rate and morbidity. Despite promising development in cancer therapy, there are still many problems (e.g. delayed diagnosis and poor prognosis) in tumor elimination that require enhancements. Currently, most clinical therapeutic targets and tumor biomarkers are proteins. However, just two percent of the human genome can translate into proteins. Hence, scientists require attention to non-coding areas, where more tumorigenic mutations happen compared to coding areas [14,15]. A large number of investigations have demonstrated the tissue specificity of several LncRNAs and dysregulated expression of them approved in cancer (Table 1) [16]. These LncRNAs with high tissue-specific properties can be collected through noninvasive methods from the circulation. It should be noted that the function of various non-coding RNAs as well as the reference and organism of studies were discussed in previous investigations (see the Luo et al., 2020; Momtazmanesh and Rezaei, 2021; and Rezaei et al., 2021 literatures). These ideal properties make lncRNAs a powerful cancer biomarker. Furthermore, the inappropriate lncRNAs expression and their pivotal function in numerous physiological procedures make them well-suited for cancer therapy. In the realm of tumorigenesis and cancer progression, non-coding RNAs (ncRNAs) exhibit a dual nature, functioning as either oncogenes or suppressors, much like microRNA (miRNA). HOTTIP, which originates from the HOXA gene, is notably upregulated in many cancer cases. Luo et al.'s research has illuminated the oncogenic properties of HOTTIP in acute myeloid leukemia (AML) [17,18].

**Table 1:**
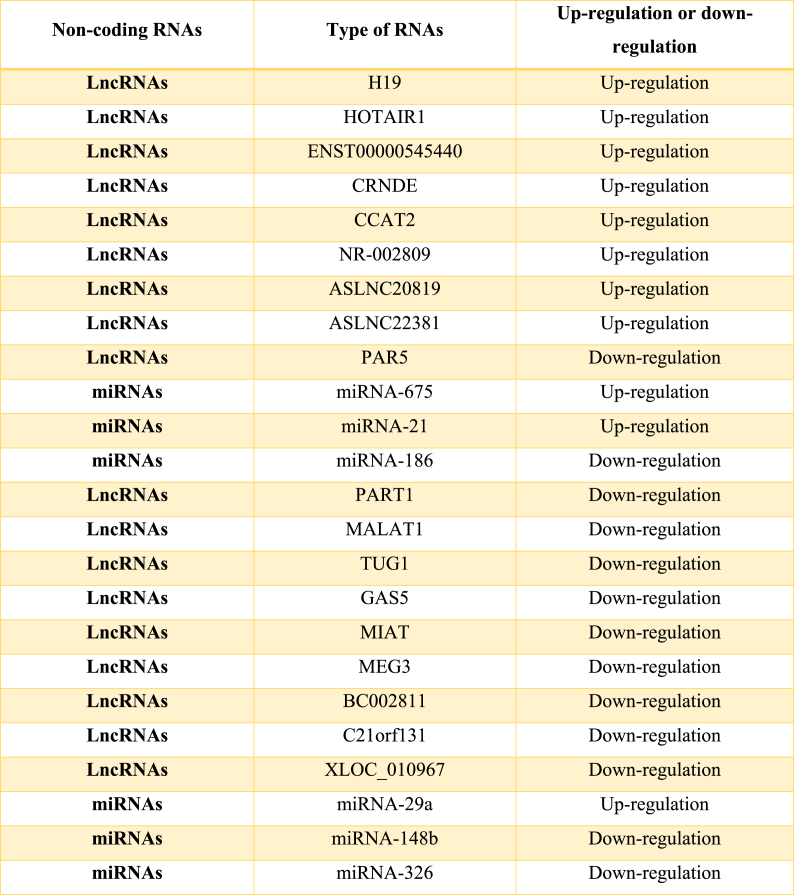
Expression levels of long non-coding RNAs (LncRNAs) and microRNAs (miRNAs) in glioblastoma.

In AML cases, it was observed that HOTTIP expression was abnormally elevated. This particular long non-coding RNA served as an epigenetic regulator, influencing the chromatin signature and transcription of genes associated with hematopoiesis. Another long non-coding RNA, LncTCF7, is transcribed from the TCF gene locus. The investigation led by researchers unveiled that lncTCF7 is upregulated in liver cancer stem cells (CSCs). and played an influential role in the cells' self-renewal process. The recruitment of the SWI/SNF complex to the TCF7 promoter by LncTCF7 is instrumental in activating Wnt signaling, which is indispensable for the continuous self-renewal of liver CSCs. Additionally, the oncogenic role of the epigenetically induced lncRNA1 (EPIC1) has been first recognized in luminal B breast cancer [[19], [20], [21]]. The EPIC1 expression has been determined to be notably elevated in glioma [[7], [8], [9], [10]], cholangiocarcinoma [22,23], pancreatic, and lung cancers [[24], [25], [26]]. Interaction between Elevated EPIC1 and MYC has a significant impact on promoting tumor growth by upregulating specific genes, including CDKN1A, CCNA2, and CDC20 [21]. Li et al. recently unveiled the role of linc0624 as a molecular decoy in the segregation of the HDAC6-TRIM28-ZNF354C transcriptional corepressor complex from specific genomic loci. This discovery sheds light on how linc0624 pays off the progression of hepatocellular carcinoma [27].

Certain lncRNAs succor as suppressors in impeding the progression and growth of cancerous cells. An isoform of the lncRNA called Pvt1b, which is dependent on the p53 protein, effectively suppresses lung cancer growth by decreasing c-Myc expression [28]. A correlation exists between reduced DIRC3 expression in melanomas and decreased overall survival [29]. Further investigation has uncovered that DIRC3 hinders the growth of melanoma cells by increasing the expression of the IGFBP5 gene, acting as a tumor suppressor. Notably, recent findings have revealed that SATB2 AS1, an antisense transcript of the tumor suppressor SATB2, is downregulated in colorectal cancer. Knockdown of SATB-AS1 leads to a notable rise in cell proliferation, migration, and invasion [[30], [31], [32]]. The heightened SATB2 then recruits HDAC1 to the Snail promoter, leading to the suppression of Snail expression and the inhibition of epithelial-to-mesenchymal transition. Additionally, MALAT1, a long non-coding RNA located in the nucleus, functions as a suppressor of tumors in cases of breast cancer. findings presented by Jong et al. indicated that the knockout of MALAT1 had a profound impact on the metastasis of breast cancer by disrupting the recruitment of the co-activator YAP and the transcription factor TEAD to the promoters of target genes [[7], [8], [9], [10],^33^].

## 3 Long non-coding RNAs in cancer

The realm of cancer research has experienced a notable shift towards lncRNAs in recent times. These lncRNAs, constituting the majority of ncRNAs, have become focal points in the field. With an estimated 102,000 lncRNAs, the extensive pool of these molecules holds significant promise for enhancing cancer treatment [34,35]. Exceeding 200 nucleotides in length, LncRNAs are non-coding transcripts that predominantly reside in the nucleus post-transcription [36,37]. Initially regarded as transcription noise Owing to their reduced expression levels, lncRNAs have now been recognized for their function in regulating transcription and post-transcription by interacting with DNA, RNA, or proteins. They either facilitate or impede transcription loop formation, and hire or inhibit regulators to modulate gene transcription [38,39]. Furthermore, the regulation of mRNA splicing is intricately influenced by lncRNAs which serve as precursors for diverse types of ncRNAs, such as miRNAs [40]. lncRNAs play a role as either tumor suppressors or oncogenes, contributing to a spectrum of signaling pathways [41].

Upon scrutinizing the expression levels of lncRNA in urine sediments, blood, and tissue specimens, a multitude of lncRNAs have been discovered, showing immense potential as supplementary or independent biomarkers in the prognosis and diagnosis of cancer [42,43]. Currently, there is a limited number of biomarkers or therapeutic agents specifically targeting lncRNAs. However, a major advancement is the discovery that the prostate cancer antigen 3 (PCA3) can be used as a diagnostic marker for prostate cancer (PCa) that currently has received approval for clinical use [44]. Furthermore, several lncRNAs are currently being evaluated in clinical trials or patented, a topic we will explore further later on. Besides, continual research on lncRNA-associated drug breakthroughs, such as SCN1ANAT in Dravet syndrome, UBE3A-ATS in Angelman syndrome, and SMN-AS1 in spinal muscular atrophy, exemplify the remarkable potential of lncRNAs [[45], [46], [47], [48]]. A deep understanding of lncRNAs is crucial for unraveling their involvement in diseases such as cancer and for harnessing their therapeutic capacity in cancer treatment. Recent research indicates that lncRNAs play a crucial part in various intracellular molecular networks. Current studies point to the significant role of lncRNAs in varied intracellular molecular networks. Their expression levels are influenced by multiple factors, and they function as regulatory elements in intricate networks. The diverse mechanisms governing these regulations can be condensed into four main types: signal, scaffold, decoy, and guide [15].

(i) The expression levels of particular lncRNAs can fluctuate across different cell states. Consequently, these lncRNAs can function as signals, providing valuable insights into the developmental or pathological status. For instance, Xist, primarily transcribed by the inactive X chromosome, can be utilized as an indicator for X chromosome inactivation.
(ii) LncRNAs are capable of binding to proteins and acting as scaffolds to facilitate the formation of regulatory complexes. for example, by engaging with polycomb repressive complex 2 (PRC2), HOTAIR can recruit EZH2 for the promotion of H3K27 trimethylation or LSD1 for the removal of H3K4me2 methylation.
(iii) Through their decoy function, lncRNAs give rise to regulating gene expression by impeding the attachment of transcription regulators. A specific example is the p53-dependent PANDA, which effectively blocks proptosis by sequestering NF-YA. Additionally, lncRNAs serve as competitive endogenous RNAs (ceRNAs) by attaching to miRNAs and halting RNA degradation, a phenomenon frequently observed in cancer. H19 serves as a ceRNA for both microRNA-152 (miR-152) in breast cancer and miR-17-5P in thyroid cancer.
(iv) lncRNAs possess the ability to direct transcription factors towards particular locations. Consequently, MEG3 acts as a guide for PRC2 and collaborates with DNA to form a complex. It is worth noting that an individual lncRNA can have one or more of these functions because they are not mutually exclusive.

## 4 Ongoing clinical trials are investigating the role of long non-coding RNAs and immunotherapies in diagnosing and treating glioma

The assessment of diagnostic and therapeutic approaches for various cancer types, including gliomas, heavily depends on clinical trials. Multiple studies have underscored the considerable potential of lncRNA in the treatment and diagnosis of different malignancies like prostate cancer (NCT03830619), hepatocellular carcinoma (NCT05088811), and lung cancer. Furthermore, ongoing clinical trials on immunotherapies for glioma, like cellular immunotherapy using intra-tumoral alloreactivity cytotoxic T lymphocytes and interleukin-2, are being conducted. However, there is a lack of clinical studies investigating the use of lncRNAs as therapeutic tools or diagnostic for glioma. Therefore, more studies are required to establish the role of lncRNA in GBM and other cancers as biomarkers and standard therapy [49].

## 5 Molecular mechanism of oncogenic long non-coding RNAs in GBM

Numerous carcinogenic lncRNAs have exhibited up-regulation in GBM specimens. One such example is MIR22HG, a cancer-causing lncRNA that has been identified as highly deregulated in GBM through the analysis of accessible datasets. MIR22HG serves as a host for miR-22-5p and miR-22-3p. Recent research has unveiled that the MIR22HG/miR-22 pathway is excessively active in both GBM and glioma stem-like cells. High levels of MIR22HG in GBM samples have been associated with unfavorable outcomes for patients. Downregulating this lncRNA has resulted in the deactivation of the Wnt/b-catenin pathway by regulating miR-22-5p and miR-22-3p expressions. In xenograft models, the suppression of MIR22HG has manifested a remarkable decrease in cell proliferation, invasion, and tumor growth. These mentioned miRNAs have been found to target PCDH15 and SFRP2 [50]. The involvement of MIR22HG in the Wnt/β-catenin signaling pathway has been widely acknowledged, highlighting its significance in this particular cancer type. Silencing MIR22HG has been suggested as a prospective therapeutic approach. Another lncRNA, small nucleolar RNA host gene 5 (SNHG5), is also elevated in GBM and is correlated with enhancing cell proliferation while inhibiting cell apoptosis. Its expression is induced by the Yin Yang 1 (YY1) transcription factor, leading to oncogenic effects through the stimulation of the MAPK/p38 axis [51,52]. The overexpression of SNHG9 in GBM samples has been associated with a decline in patient survival rates. SNHG9 has a hand in repressing the expression of miR-199a-5p and promoting the expression of Wnt2 in GBM cells. Moreover, this long non-coding RNA is confirmed to augment aerobic glycolysis and stimulate cell proliferation [11,12,53].

In patients diagnosed with GBM, there has been a notable increase in the expression of SAMMSON in their plasma, whereas no such increase was found in individuals with diffuse neurosarcoidosis, despite the similarity in MRI findings between the two conditions. Evidence suggests that this lncRNA is responsible for suppressing miR-622 in GBM cells, resulting in heightened cell proliferation [[54], [55], [56], [57]]. GBM exhibits an increased expression of MIAT lncRNA. To investigate its role, Bountali et al. conducted an experiment where they downregulated MIAT in GBM cell lines and analyzed the RNA profile using RNA sequencing. The results demonstrated differential expression of various genes that participated in cancer-associated processes, such as reactive oxygen species generation, cell growth, viability, apoptosis, and migration. Functionally, the silencing of MIAT resulted in the cessation of durable viability and migration, while promoting apoptosis in these cells [58,59]. Genome-wide expression analysis in GBM cells revealed that MALAT1 significantly upregulated post-temozolomide (TMZ) treatment. The expression of this lncRNA is modulated by p53 and p50 via kB- and p53-binding sites situated in its coding sequence. Knocking down MALAT1 escalated the responsiveness of patient-derived GBM cells to TMZ and enhanced the drug's effectiveness in xenograft mouse models [50,60]. UCA1 is an additional cancer-causing lncRNA that boosts cell migration and growth while inhibiting cell apoptosis. By interacting with miRNA, transcription factors, and an array of molecules, LncRNA exerts its regulatory control over a wide range of biological processes. These interactions have a significant impact on the modulation of proliferation, autophagy, apoptosis, migration, invasion, vasculogenic mimicry (VM), epithelial-mesenchymal transition (EMT), and various other biological behaviors [61,62]. The development and advancement of cancerous tumors are accompanied by the abnormal expression of lncRNA HULC. Previous investigations have consistently demonstrated an upregulation of lncRNA HULC expression in ovarian cancer, colon cancer, liver cancers, and other tumor tissues. Additionally, this upregulation has shown a positive correlation with TNM stage, tumor size, chemotherapy resistance, and an unfavorable prognosis [63,64]. Thus, lncRNA HULC serves as an oncogenic element that expedites the advancement of different kinds of malignant tumors. previous investigation demonstrated a notable upregulation of lncRNA HULC expression in the malignant behavior of GBM (GBM) in humans. Bioinformatic analysis suggested that elevated HULC levels in their GBM tissues correlated with poorer prognosis and the HULC overexpression stimulated the migration, invasion, and proliferation of human GBM U87 cells by surging the HIF-related EGFR/PI3K/AKT signaling pathway [[65], [66], [67], [68], [69]]. GBM stem cell-like cells rely on the EGFR/PI3K/AKT signaling cascade for their function to create vasculogenic mimicry (VM). Activation of PI3K triggers the expression of MMP14 and the maturation of MMP2, which is essential for the promotion of VM in solid GBM and the development of vascular basement membrane [42,43,68]. Yin et al.'s study explored how changes in lncRNA HULC expression affect MMP expression and tubular formation in GBM cells by silencing GBM cells and creating stable HULC overexpressing. Results showed that HULC overexpression increased tubular structures in U87 cells and SHG44, while HULC silencing diminished them. In both in vitro and in vivo GBM cells, the expression of MMP2 and MMP9 was increased when HULC was overexpressed. Conversely, the silencing of HULC led to a subside in MMP9 and MMP2 expression in these cells. These findings recommend that the lncRNA HULC might improve VM in GBM by increasing the expression of MMP2 and MMP9, offering potential therapeutic targets for GBM progression [70]. LncRNAs are commonly acknowledged to impact cancer characteristics by regulating the target gene expression through different mechanisms, including genomic imprinting, chromatin modification, sequestering miRNAs, and RNA decay [71]. Up to now, an increasing amount of lncRNAs have been discovered as crucial regulators in the control of drug resistance, genomic instability, apoptosis, cell proliferation, and tumor progression in different types of malignant tumors like GBM. A previous study revealed that in anaplastic glioma, AGAP2-AS1 expression correlated with tumor grade, and blocking AGAP2-AS1 impeded cancer cell growth in vitro [72,73]. AGAP2-AS1 expression was found to be elevated in both GBM regions. Elevated level of AGAP2-AS1 was linked to a negative clinical outcome in people with glioma, as reported by scientists. To gain a more comprehensive understanding of AGAP2-AS1 in GBM, loss- and gain-of-function assays were conducted. The results demonstrated that in U251/MG and U87/MG cells, and also down-regulated AGAP2-AS1 exhibited inhibitory effects on proliferation and invasion, while concurrently promoting apoptosis. Conversely, AGAP2-AS1 overexpression produced contrasting effects in A172 cells. These findings are accordant with the study conducted by Li et al., which revealed that increased levels of AGAP2-AS1 were observed to stimulate the proliferation, migration, and invasion of NSCLC cells while inhibiting their apoptosis. Moreover, Qi et al. presented compelling evidence demonstrating the role of AGAP2-AS1 overexpression in promoting invasion and cell proliferation in gastric cancer. Similarly, researchers reported a significant correlation between breast cancer growth and AGAP2-AS1, as well as resistance to trastuzumab. Furthermore, a recent report verified that inhibition of AGAP2-AS1 resulted in decreased migration, invasion, and cell growth, as well as increased apoptosis in GBM. However, the AGAP2-AS1-related regulatory mechanisms remain mysterious in glioma conditions [[74], [75], [76]]. It is known that lncRNAs can recruit RNAs or proteins to specific genes, thereby indirectly influencing biological processes. To shed light on the potential mechanism of AGAP2-AS1 in GBM pathogenesis, subcellular fractionation assays were carried out. The results demonstrated that AGAP2-AS1 is predominantly localized in the nucleus fractions of U251/MG and U87/MG cells, suggesting its involvement in transcriptional regulation. Further analysis through RIP and RNA pull-down assays confirmed the direct interaction between AGAP2-AS1 and EZH2 as well as LSD1. Aberrant overexpression of EZH2 and LSD1 is tied to various cellular functions in different forms of cancers [77,78]. Through the use of Western blot and qRT-PCR analysis, it has been discovered that the protein and mRNA levels of TFPI2 increase in U87/MG and U251/MG cells when AGAP2-AS1 is knocked down. Suppression of LSD1 and EZH2 in U87/MG and U251/MG cells was associated with an augmentation in TFPI2 mRNA and protein levels. This indicates that TFPI2 is a new target of AGAP2-AS1 in GBM cells. LSD1 acts as a negative regulator through histone 3 lysine 4 demethylation (H3K4me2), while EZH2 functions as a negative regulator of transcription through histone 3 lysine 27 trimethylation (H3K27me3). Further ChIP assays have revealed that AGAP2-AS1 can engage EZH2 and LSD1 to the promoter region of TFPI2, leading to the suppression of their transcription by mediating H3K4me2 and H3K27me3 modifications. In conclusion, by binding to EZH2 and LSD1, AGAP2-AS1 plays a crucial role in the epigenetic inhibition of TFPI2 expression in GBM cells. These findings have demonstrated the oncogenic functions of AGAP2-AS1 in NSCLC, gastric cancer, and breast cancer, where the involvement of epigenetic proteins like EZH2, LSD1, and CREB-binding protein (CBP) enables it to effectively suppress the transcription of downstream targets. These discoveries indicate that AGAP2-AS1 expression is amplified in both GBM tissues and cells. By acting as a scaffold for LSD1 and EZH2, AGAP2-AS1 contributes to the silencing of TFPI2 expression. Functionally, knocking down AGAP2-AS1 has been shown to impede proliferation and invasion, while promoting apoptosis in GBM cells. Through epigenetic procedures, AGAP2-AS1 contributes to the oncogenic activity of GBM, providing valuable insights into the tumorigenesis of GBM and presenting a hopeful molecular target for patients with GBM. However, additional research is necessary to investigate alternative regulatory systems of AGAP2-AS1 involved in GBM progression [17,18]. Despite the weak evolutionary conservation seen in most lncRNAs, the nuclear lncRNA MALAT1 (metastasis-associated lung adenocarcinoma transcript 1) stands out for its exceptional conservation and abundance in benign tissues. Extensive research from clinical-epidemiological studies and cell culture experiments consistently indicate that MALAT1 plays a role as a tumor promoter in GBMs, with its inhibition resulting in reduced tumor growth in a large number of preclinical GBM models.

The involvement of MALAT1 in the stemness of GBM cells is mediated through the regulation of key factors such as nestin, SOX2, and members of the WNT pathway. Its impact on GBM cells is multifaceted, as it induces protective autophagy and suppresses apoptosis by sequestering miR-101. Additionally, MALAT1 contributes to the development of chemoresistance to temozolomide by upregulating thymidylate synthase, promoting epithelial-mesenchymal transition, and suppressing miR-203. Interestingly, blocking MALAT1 expression enhances the permeability of the blood-brain tumor barrier by miR-140, which in turn facilitates the distribution of chemotherapy agents into GBM tissues. However, conflicting evidence suggests that MALAT1 in GBMs may act as a tumor suppressor by suppressing MMP2 and ERK/MAPK signaling and repressing miR-155, leading to an increase in FBXW7 expression. Regardless of its dual nature, MALAT1 appears to be a significant contributor to GBM pathogenesis. Thus, further studies utilizing diverse models, including primary GBM cultures, transgenic mice, and orthotopic and xenograft in vivo models are warranted to achieve a comprehensive grasp of its role [[7], [8], [9], [10],^79^]. In recent times, an increasing amount of research dedicated to understanding the effects of MALAT1 on GBM pathogenesis. However, the available data on MALAT1's actions in gliomas seems to present conflicting findings. Notably, the two studies showing the anti-tumorigenic function of MALAT1 in GBM relied on established cell lines (SHG139, U87, U251), while the more recent studies highlighting its pro-tumorigenic functions were executed using patient-derived primary GBM cultures [[80], [81], [82]]. The loss of inherent molecular and pathophysiological features in established cell lines is a well-known phenomenon, which can lead to conflicting results due to suboptimal cell culture conditions. The pro-tumorigenic functions of MALAT1 have been observed in studies conducted on established cell lines. This suggests that the actions of MALAT1 may not be dual in nature. However, to gain a more precise understanding of MALAT1's functions, further research using primary GBM cultures, particularly through 3-dimensional spheroid culture experiments, is necessary. Therefore, numerous new studies utilizing different primary GBM cultures, orthotopic, and xenograft in vivo models, and transgenic mice are required in the near future. If it is proven that MALAT1 promotes tumor growth, its suppression could also lead to increased permeability of the blood-tumor barrier, allowing for better delivery of anticancer chemotherapy agents. Currently, diverse therapeutic strategies are being pursued to specifically target lncRNAs, and several pharmaceutical companies are actively involved in the development of lncRNA-targeting therapeutics [83,84]. Ribozymes, deoxyribozymes, or antisense oligonucleotides (ASOs) can be tailored to target lncRNAs in cases where siRNAs are not ideal due to nucleotide sequence or secondary structure limitations. ASOs offer advantages over siRNAs such as high specificity, lack of dependence on RISC machinery, and minimal off-target effects. Future advancements in nano-formulations may enhance the delivery of MALAT1-targeting agents to brain tumors, underscoring the significance of investigating and targeting MALAT1 in GBM pathogenesis [79]. Recent findings suggest that lncRNAs prompt the onset and advancement of cancer, acting as either tumor suppressors or oncogenes. Disrupted levels of lncRNAs may be linked to tumor progression. A recent study highlighted the regulatory role of lncRNA PVT1 in the proliferation, invasion, and tumor growth of breast cancer cells in an orthotopic xenograft model. Moreover, the downregulation of SNHG5 was found to inhibit gastric cancer progression via the miR-32/KLF4 signaling pathway. Additionally, the interaction between lncRNA BC032469 and miR-1207-5p, as well as hTER T, was observed to promote the proliferation of cancer cells. Furthermore, NBAT1 exhibited dual roles in ovarian cancer, inhibiting proliferation and promoting apoptosis, while also influencing the metastasis and proliferation of glioma cells [55,85]. More investigation is imperative to elucidate the specific roles of lncRNAs. Recent findings reveal that lncRNA MEG3 functions as a tumor suppressor in different cancer types. Reduced levels of MEG3 have been detected in cervical cancer, influencing cancer cell growth via miR-21. Furthermore, MEG3 expression was diminished in endometrial carcinoma, breast cancer, and prostate cancer. The downregulation of MEG3 is correlated with enhanced cancer cell proliferation and potentially poor prognosis [[86], [87], [88]]. According to these findings, a notable decrease in MEG3 levels in glioma tissues and cells has been demonstrated. Moreover, the upregulation of MEG3 was found to effectively suppress the proliferation, migration, and invasion of glioma cells. Previous research has underlined the involvement of diverse miRNAs in the development of glioma, suggesting their potential roles as oncogenic or tumor suppressive factors through the modulation of gene expression, making them crucial targets for lncRNAs. Furthermore, the impact of miR-93 on the malignant behavior of human glioma cells and its contribution to chemoresistance were demonstrated. To shed light on the underlying mechanisms, a series of experiments were conducted to recognize the downstream molecules regulated by MEG3 in glioma. The findings unveiled that MEG3 directly interacted with miR-96-5p, which exhibited a significant upregulation in both glioma tissues and cells. Moreover, MEG3 was found to suppress the miR-96-5p expression in glioma cells, whose expression levels were tied to glioma tissues and cells. Furthermore, the inhibition of miR-96-5p led to a notable suppression of glioma growth and metastasis. Remarkably, these results align with previous studies, highlighting the role of MEG3 in impeding bladder urothelial carcinoma progression through the modulation of the miR-96 signaling pathway [[54], [55], [56],^89^]. A previous study has provided evidence that miR-96 plays a significant role in promoting transitional cell carcinoma by modulating cell apoptosis through FOXO1. Moreover, miR-96-5p has been identified as a direct regulator of MTSS1. Further functional analysis has revealed that MTSS1 is downregulated in both glioma cells and tissues. Decreased expression of MTSS1 can be achieved through miR-96-5p mimics and reinstated by overexpressed MEG3. MTSS1 is present in various tissues, including the thymus, prostate, and spleen. However, it is downregulated in different sets of tumors, such as bladder cancer, gastric, and breast, which is tied to a lower survival rate [[30], [31], [32],^90^,^91^]. The combined findings have provided reasonable insights into the pivotal roles played by the MEG3/miR-96-5p/MTSS1 axis in controlling the progression of glioma.

Some lncRNAs play a critical role in tumor development. The abnormal expression patterns of specific lncRNAs in tumors are crucial for cancer diagnosis, prognosis, and treatment. The focus on lncRNA in tumor research is increasing, with lncRNA-HOTAIR being an example that can stimulate breast cancer metastasis through interactions with the polycomb repressive complex. The findings from Yang et al.'s research reveal that the elevated levels of lncRNAs in hepatocellular carcinoma, along with their reduced levels in tumors, have a significant influence on the development of this cancer. The PVT1 gene is commonly detected in tumors and demonstrates co-expression with other genes. This is exemplified by Nagoshi's findings on fusion genes involving PVT1-neurobeachin and WW domain-containing oxidoreductase in multiple myeloma. Literature indicates that lncRNA-PVT1 is highly expressed in gastric cancer, ovarian cancer, breast cancer, prostate cancer, and lung cancer [[54], [55], [56],^92^]. The exact function of increased lncRNA-PVT1 expression in tumor cells remains ambiguous. This study signified that lncRNA-PVT1 contributes to the proliferation and invasion of tumor cells. earlier findings have indicated that upregulation of miR-200a can enhance U87 cell invasion, whereas downregulation of miR-200a can impede U87 cell invasion [93,94]. The role of miR-200a in glioma invasion was suggested, along with its potential as a key miRNA in regulating glioma cell migration. the frequent overexpression of lncRNA-PVT1 in glioma tissue is proved, pointing towards its carcinogenic activity. Furthermore, inhibiting the expression of lncRNA-PVT1 in glioma cells provided additional evidence of its functional importance. multiple recent studies have provided evidence of dysregulation in the expression of the miR-200 family in various tumor tissues. These dysregulations play a role in the regulation of cellular processes, tumor cell proliferation, migration, and invasion. While the targeting of protein-coding genes by miR-200a has been well-documented, a recent study suggests that it also targets lncRNA-PVT1. The clinical glioma tissue analysis has uncovered a noteworthy inverse relationship between the expression of miR-200a and lncRNA-PVT1. This suggests that within glioma tissue, the expression of MiR-200a is capable of repressing the expression of lncRNA-PVT1 [[54], [55], [56],^95^]. The discovery of lncRNA TTTY14 has revealed its potential as both a novel oncogene and a biomarker in Testicular germ cell tumors (TGCT). It is believed that TTTY14 could potentially impact the tumor immune microenvironment and play a role in the sensitivity of TGCT drugs. Nevertheless, it is crucial to emphasize that the exact causality between TTTY14 and these observations remains unproven. To gain a comprehensive realization of the mechanisms underlying TTTY14's involvement in immune dysfunction, tumorigenesis, and drug sensitivity in TGCT, further research is imperative [4]. By modulating AKT phosphorylation, LNC00467 played a pivotal role in amplifying the invasion of TGCT cells [96]. The expression of TTTY14 demonstrated a bimodal distribution, showing reduced expression in samples obtained from fertile patients and markedly elevated expression in samples obtained from infertile patients [97]. Despite the differential expression of TTTY14 in certain tumors, its specific function and mechanism of action remain elusive. To address this knowledge gap, a study employed a comprehensive approach, leveraging high-throughput data sourced from public databases to conduct thorough exploration and validation in the field of cell biology. It was observed that the growth of testicular germ cell tumors (TGCT) was notably suppressed in vitro by inhibiting TTTY14 through knockdown. Additionally, it is identified that both DNA methylation and copy number variation facilitate the regulation of TTTY14 expression. Interestingly, the high expression of TTTY14 in intratubular germ cell neoplasm (IGCN) may indicate a dedifferentiated state similar to that seen in intratubular germ cell neoplasia of unclassified type (IGCNU). The findings point to the possibility of IGCN evolving into a more aggressive germ cell tumor [98]. TTTY14 is expressed in normal testis and plays a part in the proliferation of early human germ cells, specifically spermatogonia. However, the lack of human germ cell lines prevents experimentally determining of pro-cellular proliferative function of TTTY14 in normal germ cells. Nevertheless, these findings indicate that TTTY14 builds up the proliferation of testicular tumor cells, leading to a significant increase in clonogenesis under abnormal conditions, suggesting that cancer cells may acquire greater stemness through TTTY14-mediated proliferation. Unlike the abundant proliferation of normal germ cells, there is no corresponding increase in cell stemness. Hence, it is proposed that TTTY14 has distinct roles in physiological and pathological cell proliferation, with its involvement in malignant cell transformation under pathological conditions [4]. HOTAIRM1 exhibits dual roles as an oncogene and tumor suppressor in different solid tumors. It acts as a tumor suppressor in hepatocellular carcinoma, colorectal cancer, head and neck tumors, and gastric cancer by forming ceRNA networks. Conversely, it functions as an oncogene in pancreatic ductal adenocarcinoma, breast cancer, and lung cancer by directly regulating HOXA1 expression. Despite being upregulated in glioma as a fetal lncRNA, the prognostic significance of HOTAIRM1 in this cancer type remains unclear. analysis of TCGA and CGGA cohorts revealed that increased HOTAIRM1 expression is independently connected with a poor prognosis in glioma patients. Functional analysis further supports the role of HOTAIRM1 in promoting malignant behaviors, aligning with recent findings, establishing it as an onco-lncRNA in glioma [99,100]. The poor prognosis of glioma, despite chemotherapy, can be attributed to the resistance exhibited towards alkylating agents. This resistance is primarily driven by EMT, a process regulated by various lncRNAs. In glioma, under hypoxic conditions, initiation of zinc finger E-box-binding homeobox 1 expression is started by the HOXA transcript located at the distal tip, leading to the promotion of EMT. This occurs through the sequestration of miR-101 [101,102]. By downregulating HOTAIRM1, a significant inhibition of EMT in glioma is evidenced by the decrease in mesenchymal cell markers and the simultaneous increase in epithelial cell markers. Conversely, increased expression of HOTAIRM1 was relevant to the limited clinical efficacy of temozolomide (TMZ) treatment, while knocking down HOTAIRM1 resulted in a lower IC50 in two glioma cell lines for TMZ. These findings propose that HOTAIRM1 could be utilized as an indicator for TMZ resistance and suggest that inhibiting it could be a favorable therapeutic strategy for glioma patients. By directly regulating the expression of HOXA1, HOTAIRM1 has been implicated in the promotion of tumor malignancy in lung cancer and glioma [22,23,103,104]. There is a positive association between HOTAIRM1 levels and HOXA family genes in CGGA and TCGA datasets. lncRNAs are known to regulate tumorigenesis through CeRNA networks that involve miRNAs and target genes [105]. The creation of a ceRNA network involved HOTAIRM1, hsa-miR-495-3p, hsa-miR-129-5p, and 13 hub genes. Validation was done through qRT-PCR and luciferase assay. Studies have shown that hsa-miR-129-5p and hsa-miR-495-3p inhibit various cellular functions and enhance drug sensitivity. The identified hub genes are closely related to tumor progression and the tumor microenvironment. For example, SPP1 (also referred to as osteopontin) is recognized for its ability to drive glioma progression through the regulation of GBM-associated macrophage infiltration. In contrast, IGFBP3 hampers the agglomeration of T cells in breast cancer. Inhibiting hsa-miR-129-5p and hsa-miR-495-3p only restored a portion of hub genes after HOTAIRM1 depletion. This suggests that HOTAIRM1 not only boosts malignancy and influences the TME in glioma by sequestering hsa-miR-495-3p and hsa-miR-129-5p but also by strengthening certain hub gene expression in a non-ceRNA manner [25,26,102]. The independent predictive value of HOTAIRM1 for glioma patient response to TMZ therapy and survival is noteworthy. A ceRNA network was formulated to shed light on the potential mechanism of HOTAIRM1. Nevertheless, a drawback is that a portion of these findings stemmed from a retrospective examination of openly accessible datasets. Consequently, further experiments are required to elucidate the molecular pathways and biological role of HOTAIRM1 in glioma. The expression of the newly identified lncRNA PTAR is closely linked to the tumor subtype and is accountable for facilitating the EMT and metastasis in ovarian cancer via the miR-101-3p/ZEB1 pathway. Knocking down LINC01234 has been found to hinder the tumor formation and proliferation of gastric cancer cells, indicating its promise as a molecular target for cancer therapy [5,6,106]. Moreover, the overexpression of lncRNA in colorectal cancer (CRC) liver metastasis (UICLM) is notably elevated, contributing to poor clinical prognosis. Nonetheless, the role and molecular process of LPPAS2 in cancer, including GBM, have yet to be fully understood [107].

**Fig. 2:**
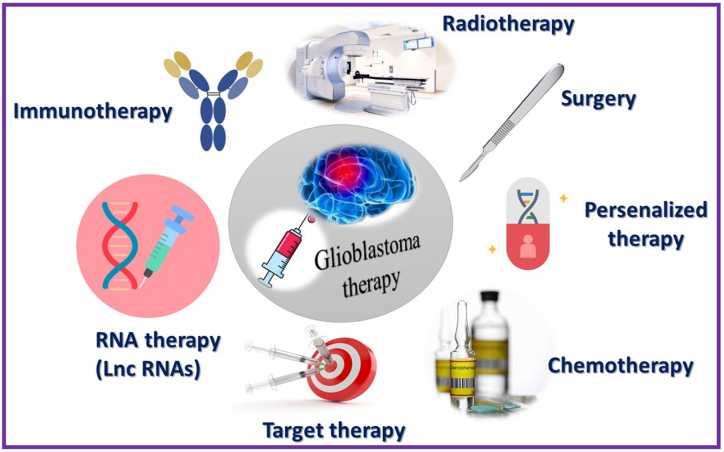
Therapeutic approaches in the treatment of GBM.

Glioma, a highly aggressive brain tumor with a grim prognosis, has not made significant progress with conventional treatments like surgery and chemotherapy (Fig. 2). As a result, there is an urgent requirement for a new effective therapeutic approach. next-generation analysis methods and integrated Gene Expression Omnibus (GEO) datasets are performed to examine the expression patterns of mRNAs and lncRNAs in GBM compared to normal brain tissues. a new lncRNA called LPP-AS2, which exhibits significantly increased expression in both GBM tissue samples and cell lines, has been discovered. This upregulation of LPP-AS2 has been considered to be strongly pertinent to a poor prognosis and unfavorable clinical survival outcomes for patients diagnosed with GBM. Through a series of gain-of-function and loss-of-function experiments, compelling evidence is provided that the presence of LPP-AS2 is known to encourage the onset and advancement of GBM, both in laboratory experiments and in living organisms. These findings highlight the carcinogenic role of LPP-AS2 and emphasize the need for further investigation into its molecular mechanisms in GBM. Analysis of differentially expressed genes from high-throughput sequencing and the development of a co-expression network revealed a positive correlation between LPP-AS2 and EGFR. EGFR, a gene established for its involvement in cell expansion and apoptosis through the activation of the PI3K/AKT signaling pathway, exhibited a direct link with LPP-AS2. Knocking down LPP-AS2 resulted in decreased EGFR expression in glioma cells, while upregulation of LPP-AS2 led to escalated EGFR expression. Additionally, LPP-AS2 influenced the levels of phospho-PI3K, phospho-AKT and c-MYC proteins [108]. In addition, functional studies have demonstrated that altering the expression levels of EGFR can partially counteract the inhibitory or stimulatory effects triggered by suppressing/elevating of LPP-AS2 in GBM cells. These observations suggest that LPP-AS2 plays a role in regulating the expression of EGFR, subsequently activating the PI3K/AKT pathway and influencing the biological characteristics of GBM cells. A growing body of research has emphasized the significance of subcellular localization in determining the molecular mechanisms of lncRNAs [109]. Additionally, a recent discovery in the realm of tumor development is the intricate interaction network involving ceRNAs. This network showcases how lncRNAs can regulate miRNA expression by competitively attaching to the intended target sites in protein-coding mRNA segments. An illustration of this phenomenon is seen with the lncRNA PDIA3P1, which changes the reaction to chemotherapy by serving as a microRNA reservoir for miR-125a/b/miR-124, resulting in increased TRAF6 expression and the amplification of the NF-κB signaling pathway [[110], [111], [112]]. The suppression of ferroptosis and the promotion of tumorigenesis in lung cancer were observed with the involvement of LINC00336 lncRNA, through its role as a competitive endogenous sponge for miR-6852 to leads an increase in cystathionine-β-synthase expression [[30], [31], [32]]. The localization of LPP-AS2 in the cytoplasm of GBM cells is a key finding, along with the observed inverse relationship with miR-7-5p as detected by RT-qPCR. These results imply that LPP-AS2 could potentially regulate gene expression post-transcriptionally. Subsequent analyses using bioinformatics tools, RNA immunoprecipitation, dual luciferase reporter assays, and RNA pulldown assays provided further confirmation of the direct binding between miR-7-5p and LPP-AS2 through complementary sequences. In addition, emerging evidence has provided substantial support for the downregulation of miR-7-5p in various cancer types, highlighting its role as a tumor suppressor. Notably, the restoration of miR-7-5p expression has been demonstrated to annihilate tumorigenesis in colorectal cancer cells and counteract the effects induced by SP1-induced lncRNA TINCR. Furthermore, miR-7-5p has shown promise as a possible indicator for small intestine neuroendocrine tumors. downregulation of miR-7-5p in GBM cells, and its overexpression effectively restrained the oncogenic behaviors associated with glioma, harmonious with recent findings. Additionally, the inhibitory effects on proliferation and invasion resulting from LPPAS2 silencing were reversed upon inhibition of miR-7-5p, while the introduction of miR-7-5p imitates partially extricated the effects caused by LPP-AS2 overexpression in GBM cells [108,113]. Overall, the collective data suggests that LPP-AS2 acts as a cancer-causing element in GBM by boosting cell survival, a process that can be partially counteracted by miR-7-5p mimics.

## 6 Tumor suppressor long non-coding RNAs

LncRNAs are involved in cancer development. Their expression in tumors is heavily influenced by epigenetic and genomic changes, allowing these lncRNAs to regulate both protein-coding and non-coding genes. Additionally, they can interact with other established cancer-related genes. LncRNAs exhibit diverse characteristics that can function as tumor suppressors, oncogenes, or have therapeutic potential. Their complex structures and capacity to participate in multicomponent complexes are believed to contribute significantly to these variations. Research indicates that lncRNAs, which show tissue- or cell type-specific gene expression, can act both as tumor suppressors and oncogenes across different cancer types [114].

Several lncRNAs have been recognized as tumor suppressors. Typically, these lncRNAs are found to be downregulated in tumor samples when compared to corresponding normal tissues. They primarily regulate the cell cycle and apoptosis; a decrease in their levels often leads to clonal expansion, heightened cell proliferation, and tumor development. Patients exhibiting higher levels of these tumor-suppressing lncRNAs tend to have more favorable clinical outcomes than those with lower expression levels. The roles of lncRNAs (tumor-suppressor features) have been validated through experiments involving the manipulation of lncRNA expression in cancer cell lines. Notable examples of such lncRNAs include GAS5, maternally expressed NKILA and gene 3 (MEG3). GAS5 is a well-studied tumor-suppressing lncRNA that is often downregulated in various cancers, including gastric and colorectal cancers as well as breast and liver cancers. It was identified as a host gene for ten small nucleolar RNAs located within its introns. The noncoding isoforms of GAS5 exert an anti-tumor effect by inhibiting tumor cell proliferation and metastasis while promoting apoptosis [115]. MEG3 is another significant tumor-suppressing lncRNA that shows reduced expression in gastric and liver cancers. Its depletion has been linked to increased angiogenesis and enhanced cell proliferation in cancer cell lines. NKILA, initially reported in the context of breast cancer, functions as a tumor suppressor by inhibiting NF-κB-mediated metastasis. Lower levels of NKILA expression are associated with poorer outcomes in breast cancer patients. In some instances, lncRNAs can be translated into stable small peptides, known as micropeptides. For instance, HOXB-AS3 encodes specific peptide (53-amino acid) that is conserved among primates. This peptide suppresses the reprogramming of glucose metabolism through inhibiting the splicing of the pyruvate kinase M gene. Another unique lncRNA, LINC00908, encodes a small regulatory peptide called ASRPS, which downregulates STAT3 phosphorylation through direct binding, thereby reducing vascular endothelial growth factor expression in triple-negative breast cancer [115]. In summary, lncRNAs can function either as tumor suppressors or oncogenes through interacting with the promoter or enhancer regions of genes, thereby influencing their expression levels.

## 7 Perspectives, challenges, and future directions

Glioma is the most prevalent malignant tumor of the central nervous system, associated with high mortality and morbidity rates. Despite significant advancements in research, the precise molecular mechanisms underlying tumor progression remain unclear, leading to a generally poor prognosis for affected patients. Over 200 lncRNAs have been identified as being linked to glioma. LncRNAs play a crucial role in regulating various cellular molecular pathways and the expression of proteins that contribute to different phases of tumor development, growth, and invasion [116]. As such, they represent promising candidates for creating innovative diagnostic, prognostic, and therapeutic strategies. The current understanding of the relationships between lncRNA expression in tissue or circulating exosomes and treatment response, as well as prognosis, raises optimism for their potential application in clinical settings. Nevertheless, despite significant progress, the function of lncRNAs in glioma is still largely unclear.

Scientists are increasingly recognizing that ncRNAs of different lengths play a variety of roles in both healthy and disease states. So far, researcher can broadly classify lncRNAs into categories such as scaffolds, sponges, guides, or peptides, which effectively capture the functions and binding behaviors of many lncRNA types. Certain lncRNAs may exhibit varying functions depending on the context, and as we deepen our understanding of the structural factors influencing these mechanisms, it will be essential to revise the existing classification system for a more precise and thorough characterization. Scientists aim to starting to recognize that the expression patterns of lncRNAs tend to be specific to certain tissues, potentially revealing new mechanisms involved in disease processes [[117], [118], [119], [120], [121], [122], [123], [124]]. This is especially evident in the brain, which contains the most diverse range of lncRNA transcripts in the body, possibly reflecting the intricate programs that determine cell fates in the nervous system. The extensive variety of ncRNAs present in the nervous system may offer a rich resource for malignant cells, allowing them to quickly exploit these elements for enhanced selective advantages and adaptability, which contribute to the aggressiveness of GBM [125]. As the scientists deepen their understanding of the molecular mechanisms that tumor cells use to promote malignant processes (e.g. spread of extrachromosomal oncogenes and the population dynamics of early genetic clones) further research will be essential to ascertain whether the dysregulated protein and DNA structures also depend on RNA components. Numerous investigations have started to highlight how the uncontrolled expression of certain lncRNAs influences cellular characteristics associated with tumor development and resistance to treatment. Nevertheless, further research is required to determine if mutations in lncRNA genes could serve as critical factors driving tumor formation or recurrence [125,126]. Consideration should also be given to lncRNA dependencies in GBM that are not linked to genetic or expression irregularities. Targeting ncRNAs essential for key cancer cell functions may overcome the challenges associated with directly targeting master regulatory proteins, which play critical roles in normal stem cell populations. This approach leverages the context-specific roles of these RNA elements. As novel technologies emerge and our knowledge of GBM advances, it increasingly becomes possible to utilize different types of therapeutic agents, making significant enhancements in the clinical treatment of this disease more achievable.

**Fig. 3:**
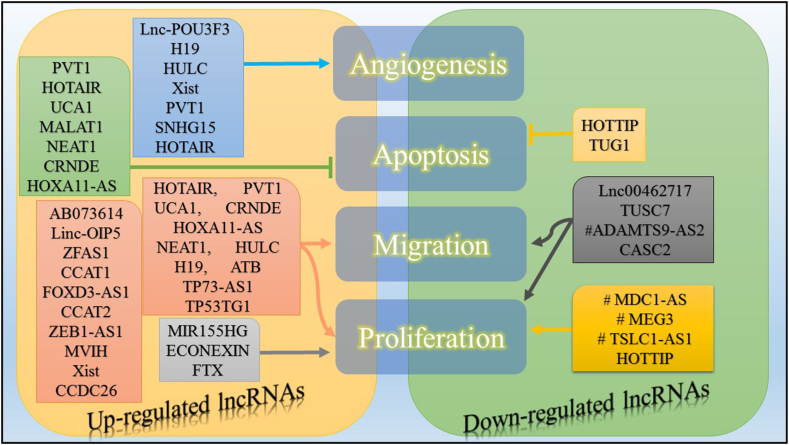
Effect of expression levels of long non-coding RNAs (LncRNAs) in GBM. Arrows show increased activity and hammerhead arrows show decreased activity; the # symbol shows poorly characterized LncRNAs.

In conclusion, GBM is a highly lethal form of brain cancer. Despite progress in surgery and radiation therapy, the need for non-invasive treatment options is crucial for improving patient outcomes. LncRNAs have garnered heedfulness in glioma research as a potential avenue for targeted therapy. The involvement of lncRNAs in assorted cellular regulatory processes influencing carcinogenesis is a well-established phenomenon. The presence of several anomalous lncRNAs has been strongly linked to glioma, influencing its various characteristics including cell proliferation, angiogenesis, motility, tumor recurrence, stemness, chemoresistance, and severity (Fig. 3). The use of cutting-edge biochemical and molecular techniques has significantly committed to our understanding of how lncRNAs control and regulate these functionalities. Although there is hope in identifying potential diagnostic tools and treatment options for improving glioma survival through lncRNAs, the majority of these molecules still possess undefined properties. The consideration of medication distribution across an intact blood-brain barrier (BBB) is crucial in the development of effective therapeutics. However, the use of lncRNA-based therapies is limited to in vivo applications owing to their limited stability and inadequate drug absorption. Therefore, it is imperative for future research to focus on identifying the dissimilarity of lncRNAs in gliomas, as this can pave the way for groundbreaking RNA-based approaches when dealing with this cancer. Moreover, such advancements would instill renewed hope in glioma patients, as recent studies have revealed that lncRNAs play a role in boosting innate immune memory responses as epigenetic regulators. Additionally, to accurately predict immune responses, it is imperative to delve into the study of immune-related lncRNAs, as they may possess the potential to be candidates for immune cell-mediated tumor evasion and cellular death.

## CRediT authorship contribution statement

**Saeid Bagheri-Mohammadi:** Writing – review & editing, Writing – original draft, Visualization, Validation, Supervision, Software, Resources, Project administration, Methodology, Investigation, Conceptualization. **Arezoo Karamivandishi:** Writing – review & editing, Visualization, Validation, Software, Investigation. **Seif Ali Mahdavi:** Writing – review & editing, Visualization, Software, Investigation. **Ali Siahposht-Khachaki:** Writing – review & editing, Visualization, Validation, Supervision, Software, Resources, Project administration, Investigation, Conceptualization.

## Data availability statement

Data sharing is not applicable to this article because no data were created or analyzed in this paper.

## Declaration of competing interest

The authors declare that they have no conflict of interest.

## References

[1] Winkle M., El-Daly S.M., Fabbri M., Calin G.A. (2021). Noncoding RNA therapeutics—challenges and potential solutions. Nat. Rev. Drug Discov..

[2] Ronco A.L., Martínez-López W., Mendoza B., Calderón J.M. (2021). Epidemiologic evidence for association between a high dietary acid load and the breast cancer risk. SciMedicine Journal.

[3] Zheng X.Y., Cao M.Z., Ba Y., Li Y.F., Ye J.L. (2021). LncRNA testis-specific transcript, Y-linked 15 (TTTY15) promotes proliferation, migration and invasion of colorectal cancer cells via regulating miR-29a-3p/DVL3 axis. Cancer Biomarkers.

[4] Cao J., Liu L., Xue L., Luo Y., Liu Z., Guo J. (2023). Long non-coding RNA TTTY14 promotes cell proliferation and functions as a prognostic biomarker in testicular germ cell tumor. Heliyon.

[5] Liang H., Yu T., Han Y., Jiang H., Wang C., You T., Zhao X., Shan H., Yang R., Yang L., Shan H. (2018). LncRNA PTAR promotes EMT and invasion-metastasis in serous ovarian cancer by competitively binding miR-101-3p to regulate ZEB1 expression. Mol. Cancer.

[6] Katoozian F., Abedi Kichi Z., Sharifi R., Shirvani-Farsani Z. (2024). The expression analysis of long non-coding RNAs related to wnt/β-catenin signaling in pancreatic cancer patients. Biochem. Genet..

[7] Wang J., Ding W., Xu Y., Tao E., Mo M., Xu W., Cai X., Chen X., Yuan J., Wu X. (2020). Long non-coding RNA RHPN1-AS1 promotes tumorigenesis and metastasis of ovarian cancer by acting as a ceRNA against miR-596 and upregulating LETM1. Aging (Albany NY).

[8] Wang J., Yang S., Ji Q., Li Q., Zhou F., Li Y., Yuan F., Liu J., Tian Y., Zhao Y., Zheng Y. (2020). Long non-coding RNA EPIC1 promotes cell proliferation and motility and drug resistance in glioma. Molecular Therapy-Oncolytics.

[9] Wang Y., Zhang Q. (2020). Long noncoding RNA MALAT1 knockdown inhibits proliferation, migration, and invasion and promotes apoptosis in non-small-cell lung cancer cells through regulating miR-515-3p/TRIM65 axis. Cancer Biother. Radiopharm..

[10] Wang S., Liu F., Ma H., Cui X., Yang S., Qin R. (2020). circCDYL acts as a tumor suppressor in triple negative breast cancer by sponging miR-190a-3p and upregulating TP53INP1. Clin. Breast Cancer.

[11] Chen X., Pan C., Xu C., Sun Y., Geng Y., Kong L., Xiao X., Zhao Z., Zhou W., Huang L., Song Y. (2019). Identification of survival-associated key genes and long non-coding RNAs in GBM multiforme by weighted gene co-expression network analysis. Int. J. Mol. Med..

[12] Chen L., Gong X., Huang M. (2019). YY1-activated long noncoding RNA SNHG5 promotes GBM cell proliferation through p38/MAPK signaling pathway. Cancer Biother. Radiopharm..

[13] Mousavi S.M., Derakhshan M., Baharloii F., Dashti F., Mirazimi S.M., Mahjoubin-Tehran M., Hosseindoost S., Goleij P., Rahimian N., Hamblin M.R., Mirzaei H. (2022). Non-coding RNAs and GBM: insight into their roles in metastasis. Molecular Therapy-Oncolytics.

[14] Bray F., Ferlay J., Soerjomataram I., Siegel R.L., Torre L.A., Jemal A. (2018). Cancer statistics 2018: GLOBOCAN estimates of incidence and mortality worldwide for 36 cancers in 185 countries. Ca-Cancer J. Clin..

[15] Qian Y., Shi L., Luo Z. (2020). Long non-coding RNAs in cancer: implications for diagnosis, prognosis, and therapy. Front. Med..

[16] Shi J., Dong B., Cao J., Mao Y., Guan W., Peng Y., Wang S. (2017). Long non-coding RNA in glioma: signaling pathways. Oncotarget.

[17] Luo H., Zhu G., Xu J., Lai Q., Yan B., Guo Y., Fung T.K., Zeisig B.B., Cui Y., Zha J., Cogle C. (2019). HOTTIP lncRNA promotes hematopoietic stem cell self-renewal leading to AML-like disease in mice. Cancer Cell.

[18] Luo W., Li X., Song Z., Zhu X., Zhao S. (2019). Long non-coding RNA AGAP2-AS1 exerts oncogenic properties in GBM by epigenetically silencing TFPI2 through EZH2 and LSD1. Aging (Albany NY).

[19] Wang Y., He L., Du Y., Zhu P., Huang G., Luo J., Yan X., Ye B., Li C., Xia P., Zhang G. (2015). The long noncoding RNA lncTCF7 promotes self-renewal of human liver CSCs through activation of Wnt signaling. Cell Stem Cell.

[20] Yan H., Bu P. (2021). Non-coding RNA in cancer. Essays Biochem..

[21] Wang Z., Cancer Genome Atlas Research N., Xie W., Yang D. (2018). lncRNA epigenetic landscape analysis identifies EPIC1 as an oncogenic lncRNA that interacts with MYC and promotes cell-cycle progression in cancer. Cancer Cell.

[22] Li Y., Cai Q., Li W., Feng F., Yang L. (2018). Long non-coding RNA EPIC1 promotes cholangiocarcinoma cell growth. Biochemical and biophysical research communications.

[23] Li Q., Dong C., Cui J., Wang Y., Hong X. (2018). Over-expressed lncRNA HOTAIRM1 promotes tumor growth and invasion through up-regulating HOXA1 and sequestering G9a/EZH2/Dnmts away from the HOXA1 gene in GBM multiforme. J. Exp. Clin. Cancer Res..

[24] Zhang B., Lu H.Y., Xia Y.H., Jiang A.G., Lv Y.X. (2018). Long non-coding RNA EPIC1 promotes human lung cancer cell growth. Biochemical and biophysical research communications.

[25] Xia P., Liu P., Fu Q., Liu C., Luo Q., Zhang X., Cheng L., Qin T., Zhang H. (2020). Long noncoding RNA EPIC1 interacts with YAP1 to regulate the cell cycle and promote the growth of pancreatic cancer cells. Biochem. Biophys. Res. Commun..

[26] Xia H., Liu Y., Wang Z., Zhang W., Qi M., Qi B., Jiang X. (2020). Long noncoding RNA HOTAIRM1 maintains tumorigenicity of GBM stem-like cells through regulation of HOX gene expression. Neurotherapeutics.

[27] Li Z., Lu X., Liu Y., Zhao J., Ma S., Yin H., Huang S., Zhao Y., He X. (2021). Gain of LINC00624 enhances liver cancer progression by disrupting the histone deacetylase 6/tripartite motif containing 28/zinc finger protein 354C corepressor complex. Hepatology.

[28] Olivero C.E., Martínez-Terroba E., Zimmer J., Liao C., Tesfaye E., Hooshdaran N., Schofield J.A., Bendor J., Fang D., Simon M.D., Zamudio J.R. (2020). p53 activates the long noncoding RNA Pvt1b to inhibit Myc and suppress tumorigenesis. Molecular cell.

[29] Coe E.A., Tan J.Y., Shapiro M., Louphrasitthiphol P., Bassett A.R., Marques A.C., Goding C.R., Vance K.W. (2019). The MITF-SOX10 regulated long non-coding RNA DIRC3 is a melanoma tumour suppressor. PLoS Genet..

[30] Wang H., Zhao Y., Cao L., Zhang J., Wang Y., Xu M. (2019). Metastasis suppressor protein 1 regulated by PTEN suppresses invasion, migration, and EMT of gastric carcinoma by inactivating PI3K/AKT signaling. J. Cell. Biochem..

[31] Wang M., Mao C., Ouyang L., Liu Y., Lai W., Liu N., Shi Y., Chen L., Xiao D., Yu F., Wang X. (2019). Long noncoding RNA LINC00336 inhibits ferroptosis in lung cancer by functioning as a competing endogenous RNA. Cell Death Differ..

[32] Wang Y.Q., Jiang D.M., Hu S.S., Zhao L., Wang L., Yang M.H., Ai M.L., Jiang H.J., Han Y., Ding Y.Q., Wang S. (2019). SATB2-AS1 suppresses colorectal carcinoma aggressiveness by inhibiting SATB2-dependent Snail transcription and epithelial–mesenchymal transition. Cancer Res..

[33] Jongchan K., Hai-Long P., Beom-Jun K., Fan Y., Zhenbo H., Yumeng W., Zhenna X., Siverly Chen Q., Zhu C., Jin Y. (2020). The oncogenic and tumor suppressive functions of the long noncoding RNA MALAT1: an emerging controversy. Front. Genet..

[34] Zhao Y., Li H., Fang S. (2016). NONCODE2016: an interactive database that aims to present the most complete collection and annotation of non-coding RNAs, especially long noncoding RNAs (lncRNAs). Nucleic Acids Res..

[35] Cuykendall T.N., Rubin M.A., Khurana E. (2017). Non-coding genetic variation in cancer. Current opinion in systems biology.

[36] Quinn J.J., Chang H.Y. (2016). Unique features of long non-coding RNA biogenesis and function. Nat. Rev. Genet..

[37] Gloss B.S., Dinger M.E. (2016). The specificity of long noncoding RNA expression. Biochimica et Biophysica Acta (BBA)-Gene Regulatory Mechanisms.

[38] Long Y., Wang X., Youmans D.T., Cech T.R. (2017). How do lncRNAs regulate transcription?. Sci. Adv..

[39] Hou T.Y., Kraus W.L. (2021). Spirits in the material world: enhancer RNAs in transcriptional regulation. Trends Biochem. Sci..

[40] Mangiavacchi A., Morelli G., Orlando V. (2023). Behind the scenes: how RNA orchestrates the epigenetic regulation of gene expression. Front. Cell Dev. Biol..

[41] Nandwani A., Rathore S., Datta M. (2021). LncRNAs in cancer: regulatory and therapeutic implications. Cancer letters.

[42] Xu Q., Ahmed A.K., Zhu Y., Wang K., Lv S., Li Y., Jiang Y. (2018). Oncogenic MicroRNA-20a is downregulated by the HIF-1α/c-MYC pathway in IDH1 R132H-mutant glioma. Biochemical and biophysical research communications.

[43] Xu T., Lin C.M., Cheng S.Q., Min J., Li L., Meng X.M., Huang C., Zhang L., Deng Z.Y., Li J. (2018). Pathological bases and clinical impact of long noncoding RNAs in prostate cancer: a new budding star. Mol. Cancer.

[44] Merola R., Tomao L., Antenucci A., Sperduti I., Sentinelli S., Masi S., Mandoj C., Orlandi G., Papalia R., Guaglianone S., Costantini M. (2015). PCA3 in prostate cancer and tumor aggressiveness detection on 407 high-risk patients: a National Cancer Institute experience. J. Exp. Clin. Cancer Res..

[45] Woo C.J., Maier V.K., Davey R., Brennan J., Li G., Brothers J., Schwartz B., Gordo S., Kasper A., Okamoto T.R., Johansson H.E. (2017). Gene activation of SMN by selective disruption of lncRNA-mediated recruitment of PRC2 for the treatment of spinal muscular atrophy. Proc. Natl. Acad. Sci. USA.

[46] Sumner C.J., Crawford T.O. (2018). Two breakthrough gene-targeted treatments for spinal muscular atrophy: challenges remain. The Journal of clinical investigation.

[47] Nilsson P.H., Johnson C., Quach Q.H., Macpherson A., Durrant O., Pischke S.E., Fure H., Landsem A., Bergseth G., Schjalm C., Haugaard-Kedström L.M. (2021). A conformational change of complement C5 is required for thrombin-mediated cleavage, revealed by a novel ex vivo human whole blood model preserving full thrombin activity. J. Immunol..

[48] Elgersma Y., Sonzogni M. (2021). UBE3A reinstatement as a disease‐modifying therapy for Angelman syndrome. Dev. Med. Child Neurol..

[49] Wu X., Yang L., Wang J., Hao Y., Wang C., Lu Z. (2022). The involvement of long non-coding RNAs in glioma: from early detection to immunotherapy. Front. Immunol..

[50] Rezaei O., Tamizkar K.H., Sharifi G., Taheri M., Ghafouri-Fard S. (2021). Emerging role of long non-coding RNAs in the pathobiology of GBM. Frontiers in oncology.

[51] Han M., Wang S., Fritah S., Wang X., Zhou W., Yang N., Ni S., Huang B., Chen A., Li G., Miletic H. (2020). Interfering with long non-coding RNA MIR22HG processing inhibits GBM progression through suppression of Wnt/β-catenin signalling. Brain.

[52] Li H., Li T., Huang D., Zhang P. (2020). Long noncoding RNA SNHG17 induced by YY1 facilitates the glioma progression through targeting miR-506-3p/CTNNB1 axis to activate Wnt/β-catenin signaling pathway. Cancer Cell Int..

[53] Feng S.G., Bhandari R., Ya L., Zhixuan B., Qiuhui P., Jiabei Z., Ni Z., Jing W., Fenyong S., Ji M., Bhandari R. (2021). SNHG9 promotes hepatoblastoma tumorigenesis via miR-23a-5p/Wnt3a axis. J. Cancer.

[54] Zhang Y., Yang G., Luo Y. (2019). Long non-coding RNA PVT1 promotes glioma cell proliferation and invasion by targeting miR-200a. Exp. Ther. Med..

[55] Zhang S., Guo W. (2019). Long non-coding RNA MEG3 suppresses the growth of glioma cells by regulating the miR-96-5p/MTSS1 signaling pathway. Mol. Med. Rep..

[56] Zhang H., Qin D., Jiang Z., Zhang J. (2019). SNHG9/miR-199a-5p/Wnt2 axis regulates cell growth and aerobic glycolysis in GBM. J. Neuropathol. Exp. Neurol..

[57] Xie J., Wang X., Liu S., Chen C., Jiang F., Mao K., Zeng F. (2019). LncRNA SAMMSON overexpression distinguished GBM patients from patients with diffuse neurosarcoidosis. Neuroreport.

[58] Bountali A., Tonge D.P., Mourtada-Maarabouni M. (2019). RNA sequencing reveals a key role for the long non-coding RNA MIAT in regulating neuroblastoma and GBM cell fate. Int. J. Biol. Macromol..

[59] Wang Y., Fu L., Lu T., Zhang G., Zhang J., Zhao Y., Jin H., Yang K., Cai H. (2021). Clinicopathological and prognostic significance of long non-coding RNA miat in human cancers: a review and meta-analysis. Front. Genet..

[60] Voce D.J., Bernal G.M., Wu L., Crawley C.D., Zhang W., Mansour N.M., Cahill K.E., Szymura S.J., Uppal A., Raleigh D.R., Spretz R. (2019). Temozolomide treatment induces lncRNA MALAT1 in an NF-κB and p53 codependent manner in GBM. Cancer Res..

[61] Misharin A.V., Cuda C.M., Saber R., Turner J.D., Gierut A.K., Haines G.K., Berdnikovs S., Filer A., Clark A.R., Buckley C.D., Mutlu G.M. (2014). Nonclassical Ly6C− monocytes drive the development of inflammatory arthritis in mice. Cell Rep..

[62] Marttila S., Chatsirisupachai K., Palmer D., de Magalhães J.P. (2020). Ageing-associated changes in the expression of lncRNAs in human tissues reflect a transcriptional modulation in ageing pathways. Mechanisms of Ageing and Development.

[63] Han J., Zhang J., Chen L., Shen B., Zhou J., Hu B., Du Y., Tate P.H., Huang X., Zhang W. (2014). Efficient in vivo deletion of a large imprinted lncRNA by CRISPR/Cas9. RNA Biol..

[64] Takahashi K., Ota Y., Kogure T., Suzuki Y., Iwamoto H., Yamakita K., Kitano Y., Fujii S., Haneda M., Patel T., Ota T. (2020). Circulating extracellular vesicle‐encapsulated HULC is a potential biomarker for human pancreatic cancer. Cancer Sci..

[65] Yan H., Tian R., Zhang M., Wu J., Ding M., He J. (2016). High expression of long noncoding RNA HULC is a poor predictor of prognosis and regulates cell proliferation in glioma. OncoTargets Ther..

[66] Hu Y., Ye S., Li Q., Yin T., Wu J., He J. (2020). Quantitative proteomics analysis indicates that upregulation of lncRNA HULC promotes pathogenesis of GBM cells. OncoTargets Ther..

[67] Yang Z., Li G., Ding C., Sun W., Zhang J. (2020). Long non-coding RNA HULC exerts oncogenic activity on papillary thyroid cancer in vitro and in vivo. Artificial cells, nanomedicine, and biotechnology.

[68] Mahiddine K., Blaisdell A., Ma S., Créquer-Grandhomme A., Lowell C.A., Erlebacher A. (2020). Relief of tumor hypoxia unleashes the tumoricidal potential of neutrophils. The Journal of clinical investigation.

[69] Soltani M., Rezaei M., Shirvani-Farsani Z., Rojhannezhad M. (2022). The emerging role of EMT-related lncRNAs in therapy resistance and their applications as biomarkers. Curr. Med. Chem..

[70] Yin T., Wu J., Hu Y., Zhang M., He J. (2021). Long non‐coding RNA HULC stimulates the epithelial–mesenchymal transition process and vasculogenic mimicry in human GBM. Cancer Med..

[71] Hanly D.J., Esteller M., Berdasco M. (2018). Interplay between long non-coding RNAs and epigenetic machinery: emerging targets in cancer?. Phil. Trans. Biol. Sci..

[72] Renganathan A., Felley-Bosco E. (2017). Long noncoding RNAs in cancer and therapeutic potential. Long Non Coding RNA Biology.

[73] Xi J., Sun Q., Ma L., Kang J. (2018). Long non-coding RNAs in glioma progression. Cancer Lett..

[74] Li W., Sun M., Zang C., Ma P., He J., Zhang M., Huang Z., Ding Y., Shu Y. (2016). Upregulated long non-coding RNA AGAP2-AS1 represses LATS2 and KLF2 expression through interacting with EZH2 and LSD1 in non-small-cell lung cancer cells. Cell Death Dis..

[75] Zheng Z., Chen M., Xing P., Yan X., Xie B. (2019). Increased expression of exosomal AGAP2-AS1 (AGAP2 antisense RNA 1) in breast cancer cells inhibits trastuzumab-induced cell cytotoxicity. Med. Sci. Mon. Int. Med. J. Exp. Clin. Res.: international medical journal of experimental and clinical research.

[76] Tian Y., Zheng Y., Dong X. (2019). AGAP2‐AS1 serves as an oncogenic lncRNA and prognostic biomarker in GBM multiforme. J. Cell. Biochem..

[77] Yin Y., Qiu S., Peng Y. (2016). Functional roles of enhancer of zeste homolog 2 in gliomas. Gene.

[78] Maiques-Diaz A., Somervaille T.C. (2016). LSD1: biologic roles and therapeutic targeting. Epigenomics.

[79] Baspinar Y., Elmaci I., Ozpinar A., Altinoz M.A. (2020). Long non-coding RNA MALAT1 as a key target in pathogenesis of GBM. Janus faces or Achilles' heal?. Gene..

[80] Han Y., Wu Z., Wu T., Huang Y., Cheng Z., Li X., Sun T., Xie X., Zhou Y., Du Z. (2016). Tumor-suppressive function of long noncoding RNA MALAT1 in glioma cells by downregulation of MMP2 and inactivation of ERK/MAPK signaling. Cell Death Dis..

[81] Xiong Z., Wang L., Wang Q., Yuan Y. (2018). Lnc RNA MALAT 1/miR‐129 axis promotes glioma tumorigenesis by targeting SOX 2. J. Cell Mol. Med..

[82] Liao K., Lin Y., Gao W., Xiao Z., Medina R., Dmitriev P., Cui J., Zhuang Z., Zhao X., Qiu Y., Zhang X. (2019). Blocking lncRNA MALAT1/miR-199a/ZHX1 axis inhibits GBM proliferation and progression. Mol. Ther. Nucleic Acids.

[83] Park J.Y., Lee J.E., Park J.B., Yoo H., Lee S.H., Kim J.H. (2014). Roles of long non-coding RNAs on tumorigenesis and glioma development. Brain tumor research and treatment.

[84] Zottel A., Šamec N., Videtič Paska A., Jovčevska I. (2020). Coding of GBM progression and therapy resistance through long noncoding RNAs. Cancers.

[85] Gong X., Huang M.Y. (2020). Tumor-suppressive function of lncRNA-MEG3 in glioma cells by regulating miR-6088/SMARCB1 axis. BioMed Res. Int..

[86] Zhang J., Yao T., Wang Y., Yu J., Liu Y., Lin Z. (2016). Long noncoding RNA MEG3 is downregulated in cervical cancer and affects cell proliferation and apoptosis by regulating miR-21. Cancer Biol. Ther..

[87] Zhang J.J., Guo S.H., Jia B.Q. (2016). Down-regulation of long non-coding RNA MEG3 serves as an unfavorable risk factor for survival of patients with breast cancer. Eur. Rev. Med. Pharmacol. Sci..

[88] Ma L., Wang F., Du C., Zhang Z., Guo H., Xie X., Gao H., Zhuang Y., Kornmann M., Gao H., Tian X. (2018). Long non-coding RNA MEG3 functions as a tumour suppressor and has prognostic predictive value in human pancreatic cancer. Oncol. Rep..

[89] Zhang L., Zhao F., Li W., Song G., Kasim V., Wu S. (2022). The biological roles and molecular mechanisms of long non-coding RNA MEG3 in the hallmarks of cancer. Cancers.

[90] Liu G., Zhao X., Zhou J., Cheng X., Ye Z., Ji Z. (2018). Long non-coding RNA MEG3 suppresses the development of bladder urothelial carcinoma by regulating miR-96 and TPM1. Cancer Biol. Ther..

[91] Lloreta J., Font-Tello A., Juanpere N., Frances A., Lorenzo M., Nonell L., de Muga S., Vázquez I., Cecchini L., Hernández-Llodrà S. (2017). FOXO1 down-regulation is associated with worse outcome in bladder cancer and adds significant prognostic information to p53 overexpression. Hum. Pathol..

[92] Han Y., Li X., He F., Yan J., Ma C., Zheng X., Zhang J., Zhang D., Meng C., Zhang Z., Ji X. (2019). Knockdown of lncRNA PVT1 inhibits glioma progression by regulating miR-424 expression. Oncology research.

[93] Yu F., Zheng Y., Hong W., Chen B., Dong P., Zheng J. (2015). MicroRNA-200a suppresses epithelial-to-mesenchymal transition in rat hepatic stellate cells via GLI family zinc finger 2. Mol. Med. Rep..

[94] Nanta R., Shrivastava A., Sharma J., Shankar S., Srivastava R.K. (2019). Inhibition of sonic hedgehog and PI3K/Akt/mTOR pathways cooperate in suppressing survival, self-renewal and tumorigenic potential of GBM-initiating cells. Mol. Cell. Biochem..

[95] Pillman K.A., Phillips C.A., Roslan S., Toubia J., Dredge B.K., Bert A.G., Lumb R., Neumann D.P., Li X., Conn S.J., Liu D. (2018). miR‐200/375 control epithelial plasticity‐associated alternative splicing by repressing the RNA‐binding protein Quaking. The EMBO journal.

[96] Bo H., Zhu F., Liu Z., Deng Q., Liu G., Li R., Zhu W., Tan Y., Liu G., Fan J., Fan L. (2021). Integrated analysis of high-throughput sequencing data reveals the key role of LINC00467 in the invasion and metastasis of testicular germ cell tumors. Cell Death Discovery.

[97] Bhat M.A., Sharma J.B., Roy K.K., Sengupta J., Ghosh D. (2019). Genomic evidence of Y chromosome microchimerism in the endometrium during endometriosis and in cases of infertility. Reprod. Biol. Endocrinol..

[98] Katabathina V.S., Vargas-Zapata D., Monge R.A., Nazarullah A., Ganeshan D., Tammisetti V., Prasad S.R. (2021). Testicular germ cell tumors: classification, pathologic features, imaging findings, and management. Radiographics.

[99] Liang Q., Li X., Guan G., Xu X., Chen C., Cheng P., Cheng W., Wu A. (2019). Long non-coding RNA, HOTAIRM1, promotes glioma malignancy by forming a ceRNA network. Aging (Albany NY).

[100] Hao Y., Li X., Chen H., Huo H., Liu Z., Chai E. (2020). Over-expression of long noncoding RNA HOTAIRM1 promotes cell proliferation and invasion in human GBM by up-regulating SP1 via sponging miR-137. Neuroreport.

[101] Zhang S., Wang W., Liu G., Xie S., Li Q., Li Y., Lin Z. (2017). Long non-coding RNA HOTTIP promotes hypoxia-induced epithelial-mesenchymal transition of malignant glioma by regulating the miR-101/ZEB1 axis. Biomed. Pharmacother..

[102] Ho K.H., Shih C.M., Liu A.J., Chen K.C. (2022). Hypoxia‐inducible lncRNA MIR210HG interacting with OCT1 is involved in GBM multiforme malignancy. Cancer Sci..

[103] Tian X., Ma J., Wang T., Tian J., Zhang Y., Mao L., Xu H., Wang S. (2018). LncRNA HOTAIRM1-HOXA1 axis down-regulates the immunosuppressive activity of myeloid-derived suppressor cells in lung cancer. Front. Immunol..

[104] Fujisaka Y., Iwata T., Tamai K., Nakamura M., Mochizuki M., Shibuya R., Yamaguchi K., Shimosegawa T., Satoh K. (2018). Long non-coding RNA HOTAIR up-regulates chemokine (C-C motif) ligand 2 and promotes proliferation of macrophages and myeloid-derived suppressor cells in hepatocellular carcinoma cell lines. Oncol. Lett..

[105] Cai J., Zhang J., Wu P., Yang W., Ye Q., Chen Q., Jiang C. (2018). Blocking LINC00152 suppresses glioblastoma malignancy by impairing mesenchymal phenotype through the miR-612/AKT2/NF-κB pathway. J. Neuro Oncol..

[106] Chen X., Chen Z., Yu S., Nie F., Yan S., Ma P., Chen Q., Wei C., Fu H., Xu T., Ren S. (2018). Long noncoding RNA LINC01234 functions as a competing endogenous RNA to regulate CBFB expression by sponging miR-204-5p in gastric cancer. Clin. Cancer Res..

[107] Chen D.L., Lu Y.X., Zhang J.X., Wei X.L., Wang F., Zeng Z.L., Pan Z.Z., Yuan Y.F., Wang F.H., Pelicano H., Chiao P.J. (2017). Long non-coding RNA UICLM promotes colorectal cancer liver metastasis by acting as a ceRNA for microRNA-215 to regulate ZEB2 expression. Theranostics.

[108] Zhang X., Niu W., Mu M., Hu S., Niu C. (2020). Long non-coding RNA LPP-AS2 promotes glioma tumorigenesis via miR-7-5p/EGFR/PI3K/AKT/c-MYC feedback loop. J. Exp. Clin. Cancer Res..

[109] Chen L.L. (2016). Linking long noncoding RNA localization and function. Trends Biochem. Sci..

[110] Salmena L., Poliseno L., Tay Y., Kats L., Pandolfi P.P. (2011). A ceRNA hypothesis: the Rosetta Stone of a hidden RNA language?. cell..

[111] Thomson D.W., Dinger M.E. (2016). Endogenous microRNA sponges: evidence and controversy. Nat. Rev. Genet..

[112] Xie C., Zhang L.Z., Chen Z.L., Zhong W.J., Fang J.H., Zhu Y., Xiao M.H., Guo Z.W., Zhao N., He X., Zhuang S.M. (2020). A hMTR4‐PDIA3P1‐miR‐125/124‐TRAF6 regulatory axis and its function in NF kappa B signaling and chemoresistance. Hepatology.

[113] Martínez-Terroba E., Dimitrova N. (2020). Long noncoding RNA amplified in lung cancer rewires cancer pathways. JCB (J. Cell Biol.).

[114] Guzel E., Okyay T.M., Yalcinkaya B., Karacaoglu S., Gocmen M., Akcakuyu M.H. (2020). Tumor suppressor and oncogenic role of long non-coding RNAs in cancer. Northern clinics of Istanbul.

[115] Park E.G., Pyo S.J., Cui Y., Yoon S.H., Nam J.W. (2022). Tumor immune microenvironment lncRNAs. Briefings Bioinf..

[116] Momtazmanesh S., Rezaei N. (2021). Long non-coding RNAs in diagnosis, treatment, prognosis, and progression of glioma: a state-of-the-art review. Frontiers in oncology.

[117] Aalijahan Hamid, Ghorbian Saeid (2019). Long non-coding RNAs and cervical cancer. Exp. Mol. Pathol..

[118] Bagheri Raana, Ghorbian Mohsen, Ghorbian Saeid (2024). Tumor circulating biomarkers in colorectal cancer. Cancer Treatment and Research Communications.

[119] Almanghadim Ghahramani, Hossein, Ghorbian Saeed, Khademi Nazanin Sadat, Soleymani Sadrabadi Mohadeseh, Jarrahi Esmaeil, Nourollahzadeh Zahra, Dastani Masomeh, Shirvaliloo Milad, Sheervalilou Roghayeh, Sargazi Saman (2021). New insights into the importance of long non-coding RNAs in lung cancer: future clinical approaches. DNA Cell Biol..

[120] Razavi Maryam, Ghorbian Saeid (2019). Up-regulation of long non-coding RNA-PCAT-1 promotes invasion and metastasis in esophageal squamous cell carcinoma. EXCLI journal.

[121] Naghashi Neda, Ghorbian Saeid (2019). Clinical important dysregulation of long non-coding RNA CCHE1 and HULC in carcinogenesis of cervical cancer. Mol. Biol. Rep..

[122] Aalijahan Hamid, Ghorbian Saeid (2020). Clinical application of long non-coding RNA-UCA1 as a candidate gene in progression of esophageal cancer. Pathol. Oncol. Res..

[123] Mousavi Zohreh, Ghorbian Saeid, Rezamand Azim, Roshangar Leyla, Jafari Behboud (2021). Evaluation of methylation at promoter regions of long non-coding RNAs in patients with acute lymphoblastic leukemia. Pharmaceut. Sci..

[124] Shams Fatemeh, Ghorbian Saeid (2019). Evaluation of prognostic usefulness of long noncoding RNA GAS5 and FAL1 in papillary thyroid carcinoma. J. Cell. Biochem..

[125] DeSouza P.A., Qu X., Chen H., Patel B., Maher C.A., Kim A.H. (2021). Long, noncoding RNA dysregulation in GBM. Cancers.

[126] Luo Y., Yang J., Yu J., Liu X., Yu C., Hu J., Shi H., Ma X. (2020). Long non-coding RNAs: emerging roles in the immunosuppressive tumor microenvironment. Frontiers in oncology.

